# Incidence of Running-Related Injuries Per 1000 h of running in Different Types of Runners: A Systematic Review and Meta-Analysis

**DOI:** 10.1007/s40279-015-0333-8

**Published:** 2015-05-08

**Authors:** Solvej Videbæk, Andreas Moeballe Bueno, Rasmus Oestergaard Nielsen, Sten Rasmussen

**Affiliations:** Department of Orthopaedic Surgery Research Unit, Science and Innovation Center, Aalborg University Hospital, Aarhus University, 18–22 Hobrovej, 9000 Aarhus, Denmark; Department of Clinical Medicine, Aalborg University, Aalborg, Denmark; Section of Sport Science, Department of Public Health, Faculty of Health Science, Aarhus University, Room 438, Dalgas Avenue 4, 8000 Aarhus, Denmark

## Abstract

**Background:**

No systematic review has identified the incidence of running-related injuries per 1000 h of running in different types of runners.

**Objective:**

The purpose of the present review was to systematically search the literature for the incidence of running-related injuries per 1000 h of running in different types of runners, and to include the data in meta-analyses.

**Data Sources:**

A search of the PubMed, Scopus, SPORTDiscus, PEDro and Web of Science databases was conducted.

**Study Selection:**

Titles, abstracts, and full-text articles were screened by two blinded reviewers to identify prospective cohort studies and randomized controlled trials reporting the incidence of running-related injuries in novice runners, recreational runners, ultra-marathon runners, and track and field athletes.

**Study Appraisal and Synthesis Methods:**

Data were extracted from all studies and comprised for further analysis. An adapted scale was applied to assess the risk of bias.

**Results:**

After screening 815 abstracts, 13 original articles were included in the main analysis. Running-related injuries per 1000 h of running ranged from a minimum of 2.5 in a study of long-distance track and field athletes to a maximum of 33.0 in a study of novice runners. The meta-analyses revealed a weighted injury incidence of 17.8 (95 % confidence interval [CI] 16.7–19.1) in novice runners and 7.7 (95 % CI 6.9–8.7) in recreational runners.

**Limitations:**

Heterogeneity in definitions of injury, definition of type of runner, and outcome measures in the included full-text articles challenged comparison across studies.

**Conclusion:**

Novice runners seem to face a significantly greater risk of injury per 1000 h of running than recreational runners.

**Electronic supplementary material:**

The online version of this article (doi:10.1007/s40279-015-0333-8) contains supplementary material, which is available to authorized users.

## Key Points

**Table:** 

‘Injuries per 1000 h of running’ is an important and useful measure of association that enables comparison of the risk of injury across studies.
Novice runners are at significantly higher risk of injury 17.8 (95 % CI 16.7–19.1) than recreational runners, who sustained 7.7 (95 % CI 6.9–8.7) running-related injuries per 1000 h of running.
More studies on ultra-marathon runners and track and field athletes are needed in order to calculate weighted estimates.

## Introduction

Running is one of the most popular and accessible sport activities worldwide [1, 2]. It can be performed with minimal equipment, and by a broad variety of people in almost every part of the world. In the US, more than 40,000,000 people run [2], and in Denmark and The Netherlands approximately 25 and 12.5 % of the population, respectively, run on a regular basis [3, 4].

Running-related injuries affect many runners. Unfortunately, the exact number of injuries is hard to identify because various studies have provided results on the prevalence and incidence of running-related injuries using different measures of association [5, 6]. To name a few, injuries have been reported as the number of injuries per 1000 km [7, 8]; proportion of injuries in a population [9]; number of injured runners per 100 runners [10]; and number of injured runners per 1000 h of running [11, 12]. The inconsistent use of such measures in the literature makes comparison of injury data difficult across studies.

Injuries per 1000 h of running was highlighted by Jakobsen et al. [12] as an important measure of association. They stated that the risk of injury must be related to the time spent running, in order to make the results from different studies comparable. This is supported in a review from 2012 by Lopes et al. [13], who emphasize that standardization of the number of injuries per hour of exposure is highly needed in running-related injury research.

In a review from 1992, van Mechelen [10] compared the incidence rates of running-related injuries across a few studies presenting such results. The results revealed an injury incidence of 2.5–12.5 injuries per 1000 h of running. Since then, many studies have reported information on running-related injuries in different types of runners per 1000 h of running—for instance, novice runners, recreational runners, ultra-marathon runners, and track and field athletes. However, no review has been published which systematically searched the literature for studies with information on the incidence of running-related injuries in different types of runners per 1000 h of running.

The purpose of the present review was to systematically review the literature for the incidence of running-related injuries in novice runners, recreational runners, ultra-marathon runners, and track and field athletes per 1000 h of running. A secondary objective was to compare the injury rates across different types of runners per 1000 h of running and include the data in meta-analyses.

## Methods

### Search Strategy

Five databases (PubMed, Scopus, SPORTDiscus, PEDro and Web of Science) were searched electronically, without restriction on date of publication, to identify studies that included data regarding running-related injury incidences per 1000 h of running. The search was performed in collaboration with a certified librarian at Aarhus University Library, Denmark. Full details of the electronic search strategy for PubMed are provided in the electronic supplementary material (ESM) Appendix S1. Additional studies and trials were identified by checking references of included full-text articles and published reviews within the running injury thematic. Full-text articles, which were not included after searching the databases, were included afterwards if they, to the authors’ knowledge, had information about injuries per 1000 h of running.

### Study Selection

The screening of eligible studies was performed by two reviewers (SV and AMB), in two steps. In step 1, all abstracts were evaluated according to pre-specified inclusion and exclusion criteria. Inclusion criteria for abstracts consisted of the following: subjects were children, novice runners, recreational runners, elite runners, cross-country runners, orienteers, and/or triathletes; the study was based on original research (prospective cohort studies and randomized controlled trials); the article was written in English or Danish; and the abstract included data regarding running-related injuries per 1000 h of running, or indicated that such data might be available in the full-text article. Exclusion criteria included the following: subjects were military or army recruits; studies in which participants were predominantly exposed to different types of sports other than running; original study designs consisted of cross-sectional studies, case–control studies, case series and case reports; and studies did not include original research, for instance reviews.

All abstracts were evaluated independently by each of the two reviewers and either included or excluded. In cases of disagreement between the two blinded reviewers (SV and AMB), a third reviewer (RON) made the final decision of selection.

In step 2, the two reviewers (SV and AMB) read all full texts included in step 1 as well as the full texts of the additional articles identified in the reference lists. The following criteria were used to finally include or exclude full-text articles. Inclusion criteria for full-text articles: must include findings from which it is possible to extract data on running-related injuries per 1000 h of running; articles without data on injuries per 1000 h of running, but containing data on the incidence of injuries per 1000 km. Exclusion criteria: studies in which participants were predominantly exposed to different types of sports other than running and, consequently, running-related injuries could not be distinguished from other sport injuries; if injuries per 1000 h were estimated per leg and not per individual; and data on injuries per 1000 h of running were missing, data on number of events and time at risk were unavailable, and the corresponding author was unable to provide these data after being contacted by e-mail.

Each reviewer (SV and AMB) processed the articles individually and, in cases of disagreement, they followed a consensus decision-making process. In cases where they did not reach a consensus, a third reviewer (RON) made the final judgment.

### Data Collection

The study characteristics of the included full-text articles were described to gain insight into the homogeneity of the study populations and definitions of running-related injuries. The following data were collected: author and year of publication; study design; type of runners; sample size used in the analysis; description of the study population; and definition of the running-related injury (Table 1). Estimates of the incidence of running-related injuries per 1000 h and per kilometres were extracted from all studies for further analysis. Three studies provided estimates of running-related injuries per 1000 h without 95 % confidence intervals (CIs) and without presenting the raw data needed to calculate these [12, 14, 15]. The corresponding authors were contacted and data were received from two of them [14, 15], which enabled the inclusion of these results in the meta-analyses.

**Table 1:** Description of studies

References, country of origin	Study design (follow-up)	Study population	Baseline characteristics	Musculoskeletal injury definition
Novice runners				
Bovens et al. [25], The Netherlands	Prospective cohort study (81 weeks)	73 Novice runners with little or no running experience	Age above 20 years. Only volunteers without persisting injuries were accepted. (58 men and 15 women)	Any physical complaint developed in relation with running activities and causing restriction in running distance, speed, duration or frequency
Bredeweg et al. [24], The Netherlands	Randomised controlled trial (9 weeks plus additional 4 weeks for 211 runners)	362 (171+191) All participants had not been running on a regular basis in the previous 12 months	Age range 18–65 years. No injury of the lower extremity within the preceding 3 months	Any musculoskeletal complaint of the lower extremity or lower back causing restriction of running for at least a week
Buist et al. [11], The Netherlands	Prospective cohort study (8 weeks)	629 Runners who had signed up for 4-mile running event. 474 novice runners who either restarted running or had no running experience. 155 recreational runners	Age above 18 years	Any musculoskeletal pain of the lower limb or back causing a restriction of running for at least 1 day
Buist et al. [20], The Netherlands	Randomized controlled trial (8 and 13 weeks)	486 Novice runners who had not been running on a regular basis in the previous 12 months^a^	Age range 18–65 years. No injury of the lower extremity within the preceding 3 months	Any self-reported running-related musculoskeletal pain of the lower extremity or back causing a restriction of running for at least 1 week (three scheduled trainings)
Nielsen et al. [26], Denmark	Prospective cohort study (12 months)	930 Novice runners who had not been running on a regular basis in the previous 12 months^a^	Healthy novice runners age range 18–65 years with no injury in the lower extremities or back 3 months preceding baseline investigation. Not participating in other sports for more than 4 h/week	Any musculoskeletal complaint of the lower extremity or back causing a restriction of running for at least 1 week
Recreational runners				
Jakobsen et al. [12], Denmark	Randomised controlled trial (12 months)	41 Recreational long-distance runners. Had all taken part in marathon races and intended to take part in at least two marathons during the year of investigation	19 Men and 2 women aged 24–56. No runner had any symptoms or objective signs of overuse injury at the start of the investigation	Any injury to the musculoskeletal system that was sustained during running and prevented training or competition
Malisoux et al. [14], Luxembourg	Prospective cohort study (22 weeks)	264 Recreational runners. Mean regularity of running^c^ in the last 12 months = 9.4–10.8	Healthy participants above 18 years old with any level of fitness	A physical pain or complaint located at the lower limbs or lower back region, sustained during or as a result of running practice and impeding planned running activity for at least 1 day
Theisen et al. [15], Luxembourg	Randomised controlled trial (5 months)	247 Recreational runners	Healthy and uninjured leisure-time runners, aged above 18 years. Participants having more than 6 accumulated months of regular training^b^	Any first-time pain sustained during or as a result of running practice and impeding normal running activity for at least 1 day
Van Mechelen et al. [28], The Netherlands	Randomised controlled trial (16 weeks)	421 Recreational runners running at least 10 km/week all year-round	Healthy, no current injury, not home from work at sick leave, not performing sport as a part of their profession	Any injury that occurred as a result of running and caused one or more of the following: (1) the subject had to stop running, (2) the subject could not run on the next occasion, (3) the subject could not go to work the next day, (4) the subject needed medical attention, or (5) the subject suffered from pain or stiffness during 10 subsequent days while running
Wen et al. [23], USA	Prospective cohort study (32 weeks)	MH group:108 recreational runners previously running a mean of 24.94 km/week^b^. However 8.3 % of these were novice runners with no running experience	Members of a running group with the purpose to prepare its members to run a marathon	Answering yes to having had “injury or pain” to an anatomic part; answering yes to having had to stop training, slow pace, stop intervals, or otherwise having had to modify training; and a “gradual,” versus “immediate”, onset of the injury or a self-reported diagnosis that is generally considered an overuse injury
Ultra marathon runners				
Krabak et al. [21], USA	Prospective cohort study (7 days)	396 Experienced runners who have completed marathon or ultraendurance events	Age range 18–64 years	A disability sustained by a study participant during the race, resulting in a medical encounter by the medical staff
Track and field athletes				
Bennell et al. [22], Australia	Prospective cohort study (12 months)	95 Competitive track and field athletes (throwers and walkers excluded)	Age range 17–26 years. Training at least three times a week, when uninjured	Any musculoskeletal pain or injury that resulted from athletic training and caused alteration of normal training mode, duration, intensity or frequency for 1 week or more
Lysholm et al. [19], Sweden	Prospective cohort study (12 months)	60 Track and field athletes. Sprinters, middle-distance runners and longdistance/marathon runners running in club and competing	Previous experience of training (7 h per week or more) varied between 1 and 32 years	Any injuries that markedly hampered training or competition for at least 1 week

*MH* mileage-hours

^a^10km total in all training sessions in the previous 12 months

^b^Miles were converted to km [17]

^c^Regular training (at least once a week)

The study populations of the included studies were categorized into one of four types of runners: novice runners; recreational runners; ultra-marathon runners; and track and field athletes. This categorization was made to enable comparison of results across studies.

Some studies reported the incidence of running-related injuries per 1000 miles [7, 8, 16] but these results were converted into running-related injuries per 1000 km using an online converter [17].

### Risk of Bias Assessment

The tool used for assessing risk of bias of the included studies was chosen after thorough consideration of the advantages and disadvantages of the available methods for evaluating bias. The studies included both prospective cohort studies and randomised controlled trials. The main purpose of this review was to measure the incidence of running-related injuries per 1000 h of running. The causes of running-related injuries were not of interest, thus minimizing the importance of methods of randomization for the quality of outcome. Quality assessment by one single tool was therefore possible for both designs. The tool used to assess the risk of bias of the included studies was a version of the Newcastle Ottawa Scale, a tool modified by Saragiotto et al. [18] to evaluate studies undertaking research on runners. The tool contains 11 criteria designed to assess the risk of bias, and uses a star rating system to indicate the quality of a study (see ESM Appendix S2 for a description of each criterion in the original version of the quality assessment tool modified for runners [18]). Certain modifications were applied to specify the tool used to assess the risk of bias on the parameter of concern in our review—the incidence of running-related injuries. Three of the 11 criteria were excluded. Item 4 was excluded because an exposed versus non-exposed cohort was irrelevant as long as the total study population was exposed to running; item 7 was excluded because it was linked to item 4; and item 11 concerned the risk of association and was removed because these measures relate to research on associations. In item 3 the wording ‘average runners in the community’ was reworded to ‘average type of runners researched’, meaning that the article received a star if the study population were representative of the type of runner (novice runners, recreational runners, ultra-marathon runners, or track and field athletes) described according to item 1. The criteria adopted to assess risk of bias were (1) description of runners or type of runner; (2) definition of the running-related injury; (3) representativeness of the exposed cohort; (4) ascertainment of exposure; (5) demonstration that outcome of interest was not present at the start of the study; (6) assessment of outcome; (7) was follow-up long enough for outcomes to occur; and (8) adequacy of follow-up of cohorts. The risk of bias assessment was carried out by two researchers (SV and AMB) in a blinded process and, in cases of disagreement, they went through a consensus-making process. Only studies with estimates on injury incidence per 1000 h were quality scored since this outcome represented the main analysis.

## Results

**Fig. 1:**
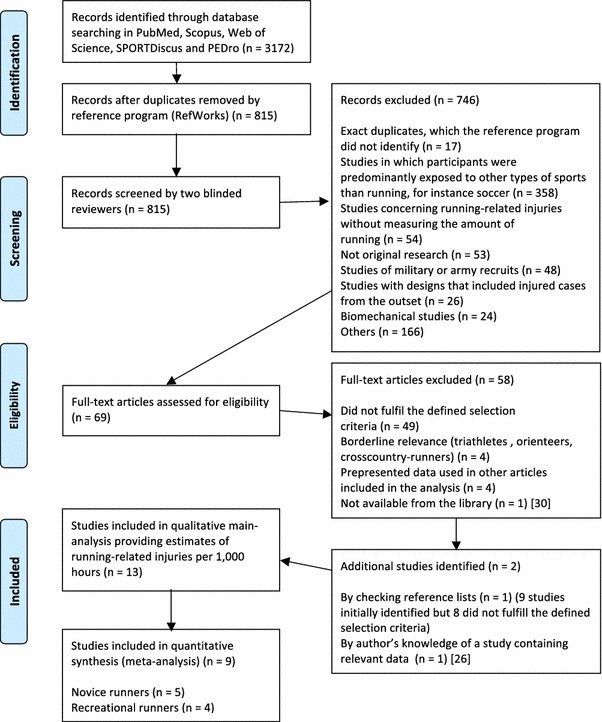
Flowchart visualizing the selection process of studies in the systematic review

A total of 3172 articles were identified through the database searches. Among these articles, 2357 were duplicates, as determined by the reference program RefWorks. Next, 815 titles and abstracts were evaluated in step one of the selection process. Of these, 69 full-text articles were included and evaluated according to the inclusion and exclusion criteria in step two of the selection process, of which 58 were excluded. In Fig. 1, the selection process is visualised in a flowchart. By checking reference lists, one additional study was included [14]. In addition, the authors knew of one article that was not included in the search but in which the relevant information was incorporated [26]. This article was also included. Finally, 13 articles that presented data on running-related injuries per 1000 h of running were included—eight prospective cohort studies and five randomized controlled trials. Overall, ten studies provided estimates on running-related injuries per 1000 km and these were used for a subanalysis.

The year of publication for the included studies ranged from 1987 to 2014, and the studies represented populations in Australia, Denmark, Luxembourg, Sweden, The Netherlands, and the USA. The follow-up periods ranged from 7 days to 81 weeks. Eight studies used a time-loss definition of injury; one study defined an injury as a need for medical attention; and the remaining four studies used a mixture of time loss, physical pain, and the need for medical attention in the definition of injury.

**Fig. 2:**
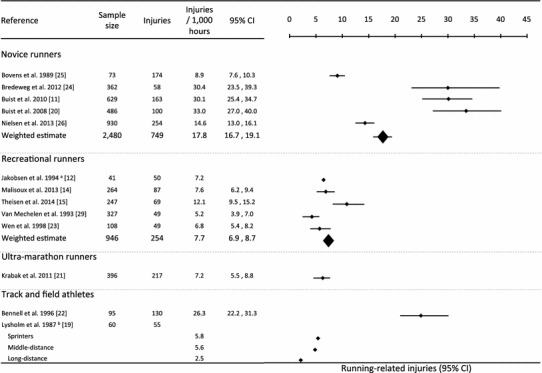
Meta-analysis performed on the estimates of running-related injuries per 1000 h in novice runners and recreational runners. ^a^Data on standard error or 95 % confidence limits were not reported and the study was therefore not included in the meta-analysis. ^b^Data on standard error or 95 % confidence limits were not reported and therefore no meta-analysis was performed on track and field athletes. *CI* confidence intervals

Across studies, the primary purpose was to compare the incidence of running-related injuries per 1000 h of running. Five studies reported this estimate in novice runners; five studies in recreational runners; one study in ultra-marathon runners; and two studies in track and field athletes. The estimates ranged from 2.5 [19] to 33.0 [20] running-related injuries per 1000 h of running. Two meta-analyses were performed on the estimates of novice runners and recreational runners, respectively. As one article [12] did not provide data to calculate 95 % CIs, estimates from nine studies were included in these quantitative analyses (Fig. 2). The weighted estimates revealed novice runners faced a significantly greater injury rate of 17.8 (95 % CI 16.7–19.1) than recreational runners, who sustained 7.7 (95 % CI 6.9–8.7) running-related injuries per 1000 h of running.

Ten studies provided estimates of running-related injuries per 1000 km of running, and these results were pooled in a subanalysis (Table 2). The weighted estimate revealed an injury incidence of 1.07 (95 % CI 1.01–1.13) per 1000 km of running.

**Table 2:** Running-related injuries per 1000 km of running

References	Runners (*n*)	Injuries (*n*)	Estimate (RRI per 1000 km)	95 % CI
Bennell et al. [22]	95	130	0.58	0.5, 0.7
Bovens et al. [25]	73	174	0.86	0.7, 1.0
Fields et al. [7]	40	17	0.18	0.1, 0.3
Gerlach et al. [8]	86	47	0.22	0.2, 0.3
Jakobsen et al. [12]	41	50	0.62	0.4, 0.9
Krabak et al. [21]	396	217	2.28	2.0, 2.6
Nielsen et al. [27]	58	13	2.85	1.7, 4.9
Nielsen et al. [26]	930	294	1.64	1.5, 1.8
Van Mechelen et al. [28]	421	49	0.44	0.3, 0.6
Wen et al. [23]	108	49	0.76	0.6, 0.9
Weighted estimate	2248	1040	1.07	1.01, 1.13

*RRI* running-related injuries, *km* kilometres, *CI* confidence interval

The risk of bias was assessed for each of the 13 studies presenting an estimate of the incidence of running-related injuries per 1000 h of running (Table 3). The criteria most frequently awarded with a star were description of runners or type of runners (13/13) and definition of running-related injury (13/13). The criteria with the least stars awarded comprised ascertainment of exposure (6/13) and assessment of outcome (8/13). The average stars awarded to the articles assessed for risk of bias was 6 out of a total of 8 stars, with a maximum of 8 and a minimum of 3.

**Table 3:** Risk of bias assessment

	Criteria for assessing risk of bias							
	1	2	3	4	5	6	7	8
Novice runners								
Bovens et al. [25]	*	*	*	0	*	*	*	*
Bredeweg et al. [24] RCT	*	*	*	0	*	0	0	0
Buist et al. [11]	*	*	*	0	0	0	0	0
Buist et al. [20] RCT	*	*	*	0	0	0	0	*
Nielsen et al. [26]	*	*	*	*	*	*	*	*
Recreational runners								
Jakobsen et al. [12] RCT	*	*	*	0	*	*	*	0
Malisoux et al. [14]	*	*	*	*	*	0	*	*
Theisen et al. [15] RCT	*	*	0	*	*	*	*	*
Van Mechelen et al. [28] RCT	*	*	*	0	*	*	*	0
Wen et al. [23]	*	*	*	0	0	0	*	*
Ultra-marathoners								
Krabak et al. [21]	*	*	*	*	0	*	0	*
Track and field athletes								
Bennell et al. [22]	*	*	*	*	*	*	*	*
Lysholm et al. [19]	*	*	*	*	0	*	*	0

Only studies providing estimates of the incidence of running-related injuries per 1000 h were assessed for risk of bias. The criteria adopted to assess risk of bias were: (1) description of runners or type of runner; (2) definition of the running-related injury; (3) representativeness of the exposed cohort; (4) ascertainment of exposure; (5) demonstration that outcome of interest was not present at start of study; (6) assessment of outcome; (7) was follow-up long enough for outcomes to occur?; (8) adequacy of follow-up of cohorts

*RCT* randomised controlled trial

* A study was awarded a star for every criterion it fulfilled. The more stars the higher quality

## Discussion

The present review is the first to systematically review the literature on the incidence rate of running-related injuries in different types of runners. The weighted estimate of 17.8 (95 % CI 16.7–19.1) running-related injuries per 1000 h of running in novice runners was significantly greater than the incidence rate of 7.7 (95 % CI 6.9–8.7) in recreational runners. One study reported the incidence of running-related injuries in ultra-marathon runners as 7.2 per 1000 h [21]. In track and field athletes, two studies reported the incidences of running-related injuries from 2.5 to 26.3 per 1000 h [19, 22]. In the latter, track and field athletes were subdivided into sprinters, middle-distance runners, and long-distance runners, which may be relevant as the reported running-related injury incidence per 1000 h was greater in sprinters and middle-distance runners than in long-distance runners [19].

In Fig. 2, a summary of the results in different types of runners is presented. The healthy participant effect may play a role when grouping novice versus recreational runners [23]. In novice runners, the five studies are heterogeneous since the estimates reported by three of the studies [11, 20, 24] range from 30.1 to 33 and are significantly higher than those reported by the two remaining studies [25, 26]. A possible explanation for the discrepancy is the follow-up time in the respective studies. The non-injured runners accumulate relatively more exposure time in studies with a long follow-up, while the injured runners are censored. This will mathematically explain the overall decrease in running-related injuries per 1000 h of running in studies with longer follow-up amongst novice runners. The two studies with the lowest incidence of running-related injuries per 1000 h of running had 81 and 52 weeks of follow-up, while the three studies with the greatest injury incidence had follow-up periods of 8–13 weeks (Table 1).

The link between a relatively short follow-up time and a high incidence rate of running-related injuries versus long follow-up time and a lower incidence rate of running-related injuries indicates the possibility that runners classified as novice runners at the beginning of a study may reasonably be classified as recreational runners as time passes. If novice runners exceed 8–13 weeks without injury, they may well have adapted to running and face a lower injury risk after this period, even though they may spend more time running. Novice runners exceeding 8–13 weeks’ follow-up may then be considered as recreational runners instead. Based on this, it may be appropriate to identify a cut-off distinguishing a novice runner from a recreational runner.

In contrast, the injury incidences are homogeneous in recreational runners and the weighted estimate is unaffected by bias.

The strengths of the present review are mainly the systematic search of the literature and the use of meta-analyses to compare the injury incidences. The searches were performed thoroughly in five databases, in cooperation with a certified librarian. Moreover, all reference lists of the included full-text articles were checked for additional studies and, to the authors’ knowledge, one article [26] was also able to be included for analysis, although it was not indexed in any of the five databases searched. Evaluation of the quality of all articles presenting estimates of running-related injuries per 1000 h was accomplished and meta-analyses on these data were conducted. Thus, the present systematic review and meta-analyses represent rigorous evaluations and provide estimates of running-related injury incidences in novice runners, recreational runners, ultra-marathon runners, and track and field athletes.

The present study has a number of limitations, including differences in definitions of injury, definition of type of runner, and outcome measures used. First, definition of injury varies considerably across studies. Eight studies used time-loss definitions, but even within this definition there is a lack of consensus of the amount of time needed to classify time loss from running as a running-related injury. One study did not define the amount of time [12], some studies used 1 day in their definition [11, 14, 15], while other studies used 1 week [19, 20, 22, 24]. The only study [21] solely defining injury as the need for medical attention was reporting on ultra-marathon runners, and as these data were collected in real time while the runners participated in the ultra-marathon, this method was reasonable. No studies exclusively defined a running-related injury as physical pain alone, but, in four studies, physical pain was incorporated as part of the injury definition [16, 22, 25, 28]. Second, runners from the included studies were classified into four groups according to the type of runner, enabling relevant intergroup comparison. No exact definition of each category was made, but the baseline characteristics leading to grouping in one of the four types of runners are listed in Table 1. Third, the method of gathering data on exposure time may be questionable. In many studies, runners were asked to self-report their training exposure in web-based running diaries. This approach may lead to training hours or distance being estimated wrongly, possibly because of recall bias and time spent self-reporting [27]. The quality assessment tool accounted for this, and awarded no star when exposure was registered by written self-report (item 5). However, it is questionable whether the risk of bias was, in reality, higher in the study by Bovens et al. [25], which received no star in item 5 because running exposure was collected in diaries, than in the study by Benell et al. [22], in which a star was awarded for a retrospective personal interview completed by one of the researchers at the end of the 12 months of follow-up. Lack of agreement in the way exposure time was calculated was another challenge. In some studies [11, 14, 15, 19, 23–25, 28], exposure time was calculated from the time a participant started the running programme until the time they reported a running-related injury (injured runners) or until the end of the programme (non-injured runners). This way of calculating exposure time was ideal due to that fact that the same runner could only contribute exposure time as long as he had not been injured. Thus, the risk of registering the same injury twice, if re-occurring, was avoided. Additionally, an injured person could not add exposure time after the injury occurred, and the number of injuries would be the same as the number of injured runners. Other studies did not mention whether study participants were censored if an injury occurred [12, 20, 26]. Further, some studies specified the premise that the same runner was included and was contributing exposure time, if running was resumed after an injury occurrence [21, 22]. Due to the varying ways of calculating exposure time in the included studies, the most appropriate comparison of the incidence of running-related injuries across all included studies was to use the total number of registered injuries instead of the total number of injured runners. This approach made it possible for one runner to figure twice or more in the pooled count of injuries. However, it would have been preferable if all studies had used the ideal method of calculating exposure time since this would have meant that one single runner could not accumulate exposure time after a first-time injury and have a recurrent injury counted twice.

Of the 13 studies providing estimates on running-related injuries per 1000 h of running, not all provided raw data on exact exposure time or 95 % CIs of the reported estimates. Corresponding authors from the respective articles [12, 14, 15, 26] were contacted, and data were received from Malisoux et al. [14], Theisen et al. [15] and Nielsen et al. [26]. Moreover, the estimate of 30.1 running-related injuries per 1000 h used in the meta-analysis relating to novice runners derives from the complete study population of runners in the prospective study of Buist et al. [11]. Overall, 155 of these 629 runners were described as runners already participating in running at baseline, running a mean of 1.2 h per week. Unfortunately, we were unable to obtain data that allowed us to calculate estimates for each of the groups of runners separately. Consequently, we decided to include the estimate of 30.1 running-related injuries per 1000 h in the category of novice runners; therefore, the true incidence of running-related injuries in novice runners might be even higher.

The present study constitutes a thorough and fully updated literature review presenting data regarding the incidence rates of running-related injuries, and outlining relevant key issues, which limit the comparison of studies in running-related injury research. The included meta-analyses form new estimates showing variations in the incidence rates of running-related injuries among different types of runners, and can be used as a starting point in future running-related injury research.

## Conclusions

The reported weighted analysis of running-related injury incidence per 1000 h of running revealed that novice runners face a significantly greater risk of injury 17.8 (95 % CI 16.7–19.1) than their recreational peers 7.7 (95 % CI 6.9–8.7). Caution is advisable when comparing estimates on the incidence of running-related injuries across studies because of differences in the definition of injury. Only a few studies reported injury incidences of ultra-marathon runners and track and field athletes, and no weighted estimates were calculated.

## Electronic supplementary material

Supplementary material 1 (PDF 62 kb)

Electronic Supplementary Material Appendix S2:The criteria adopted to assess risk of bias in all the included articles in the paper: (1) description of runners or type of runner; (2) definition of the running-related injury; (3) representativeness of the exposed cohort; (4) ascertainment of exposure; (5) demonstration that outcome of interest was not present at start of study; (6) assessment of outcome; (7) was follow-up long enough for outcomes to occur?; (8) adequacy of follow-up of cohorts (PDF 131 kb)
