# Supplementary material for: Incidence of Running-Related Injuries Per 1000 h of running in Different Types of Runners: A Systematic Review and Meta-Analysis
Source: Sports Med. 2015 May 8;45(7):1017–26. doi: 10.1007/s40279-015-0333-8 (PMC4473093; doi:10.1007/s40279-015-0333-8)
Supplement: Supplementary file 1 — Supplementary material 1 (PDF 62 kb) [file 40279_2015_333_MOESM1_ESM.pdf]

Electronic Supplementary Material Appendix S1

Specification of the search strategy used in the Pubmed database:

(488 HITS, 23th of june, 2014)

(((("Running"[Mesh]) AND (((("Athletic Injuries"[Mesh]) OR running injur*) OR running-related injur*) OR "1000 hours")) NOT ((((((((((((((((((((((((("Addresses"[Publication Type]) OR "Bibliography"[Publication Type]) OR "Biography"[Publication Type]) OR "Case Reports"[Publication Type]) OR "Clinical Conference"[Publication Type]) OR "Comment"[Publication Type]) OR "Congresses"[Publication Type]) OR "Dictionary"[Publication Type]) OR "Directory"[Publication Type]) OR "Editorial"[Publication Type]) OR "Festschrift"[Publication Type]) OR "Government Publications"[Publication Type]) OR "Interview"[Publication Type]) OR "Lectures"[Publication Type]) OR "Legal Cases"[Publication Type]) OR "Legislation"[Publication Type]) OR "Letter"[Publication Type]) OR "News"[Publication Type]) OR "Newspaper Article"[Publication Type]) OR "Retracted Publication"[Publication Type]) OR "Retraction of Publication"[Publication Type]) OR "Review"[Publication Type]) OR "Scientific Integrity Review"[Publication Type]) OR "Technical Report"[Publication Type]) OR "Validation Studies"[Publication Type])) NOT "Soccer"[Mesh]) NOT "Football"[Mesh] Filters: Danish; English
