# Supplementary material for: Incidence of Running-Related Injuries Per 1000 h of running in Different Types of Runners: A Systematic Review and Meta-Analysis
Source: Sports Med. 2015 May 8;45(7):1017–26. doi: 10.1007/s40279-015-0333-8 (PMC4473093; doi:10.1007/s40279-015-0333-8)
Supplement: Supplementary file 2 — Electronic Supplementary Material Appendix S2:The criteria adopted to assess risk of bias in all the included articles in the paper: (1) description of runners or type of runner; (2) definition of the running-related injury; (3) representativeness of the exposed cohort; (4) ascertainment of exposure; (5) demonstration that outcome of interest was not present at start of study; (6) assessment of outcome; (7) was follow-up long enough for outcomes to occur?; (8) adequacy of follow-up of cohorts (PDF 131 kb) [file 40279_2015_333_MOESM2_ESM.pdf]

Table 1 Description of the 11 criteria designed to assess risk of bias in the studies[a]

Criterion	Description of criteria
1. Description of runners or type of runners	There are several types of runners (recreational, elite, ultra marathoners, marathoners, etc.). Without the description regarding to the type of runners it is impossible to conclude which population the incidence rates refer to. Studies that reported a description of the runners or informed the type of runners receive a star for this criterion. Studies conducted in running races (which may determine the type of runners; e.g., marathon race) and which describe the race characteristics receive a star for this criterion as well. Studies that did not describe the characteristics or the type of runners, and studies conducted in running races that did not describe the characteristics of the race did not receive a star for this criterion
2. Definition of running-related musculoskeletal injury	Studies that aimed to investigate running injuries should present a definition of a running-related musculoskeletal injury informing what was considered as an injury in the study. Studies that present a definition of running-related musculoskeletal injury received a star for this criterion
3. Representativeness of the exposed cohort	(a) Truly representative of the average runners in the community*; (b) somewhat representative of the average runners in the community*; (c) selected group of users; (d) no description of the derivation of the cohort
4. Selection of the non-exposed cohort	(a) Drawn from the same community as the exposed cohort*; (b) drawn from a different source; (c) no description of the derivation of the non-exposed cohort
5. Ascertainment of exposure	(a) Secure record*; (b) structured interview*; (c) written self report; (d) no description
6. Demonstration that outcome of interest was not present at start of study	(a) Yes*; (b) no. Studies that described that all runners included were injury-free at baseline received a star for this criterion
7. Comparability of cohorts on the basis of the design or analysis	(a) Study controls for the most important factor (stated in the background of the study*; (b) study controls for any additional factor*. For this criterion, studies could be awarded with two stars
8. Assessment of outcome	(a) Independent blind assessment*; (b) record linkage*; (c) self-report; (d) no description
9. Was follow-up long enough for outcomes to occur?	(a) Yes*; (b) no. Studies that carried out a follow-up period of at least 12 weeks received a star for this criterion
10. Adequacy of follow-up of cohorts	(a) Complete follow-up of all subjects accounted for*; (b) subjects lost to follow-up unlikely to introduce bias (up to 20 % loss) or description provided of those lost*; (c) follow-up rate <80% and no description of those lost; (d) no statement. A loss to follow-up greater than 20 % may increase the risk of bias in prospective studies [21]
11. Statistic measurement for risk association	Prospective studies should inform a statistical measure to determine risk association (e.g., hazard ratio, odds ratio, relative risk) and the confidence interval. Studies that gave a statistical measure of risk received a star for this criterion

[a] The articles could be awarded a maximum of one star for each item, except for item 7, which could be awarded two stars. A total of 12 stars could be given for the articles

* Articles with this alternative received a star for this criterion
